# A pathological complete response by chemotherapy with S-1 and oxaliplatin for a locally advanced duodenal adenocarcinoma in Lynch syndrome: a case report

**DOI:** 10.1186/s40792-019-0712-8

**Published:** 2019-10-21

**Authors:** Satoshi Yasuda, Suzuka Harada, Akinori Tsujimoto, Satoko Aoki, Takeshi Takei, Kazuhiro Migita, Masato Ueno, Mitsutoshi Tatsumi, Akihiko Watanabe

**Affiliations:** Department of Surgery, Nara Prefecture Western Medical Center, 1-14-16 Mimuro Sango-cho, Ikoma-gun, Nara, 636-0802 Japan

**Keywords:** Advanced duodenal adenocarcinoma, Chemotherapy, Pathological complete response, Lynch syndrome, S-1, Oxaliplatin

## Abstract

**Background:**

Although primary duodenal adenocarcinoma (DA) is a rare malignancy representing ~ 0.5% of all gastrointestinal cancers, the incidence of DA is more frequent in Lynch syndrome. Because of its rarity, treatment strategies or optimal chemotherapeutic regimens have not been clearly defined for advanced DA.

**Case presentation:**

A 72-year-old woman with Lynch syndrome visited our hospital with a right upper abdominal pain. Computed tomography (CT) showed wall thickness with enhancement in the second portion of the duodenum and adjacent abdominal wall, which suggested direct tumor invasion to the abdominal wall. Upper gastrointestinal endoscopy (UGE) showed a large ulcerative tumor in the second portion of the duodenum, and histological analysis revealed a poorly differentiated adenocarcinoma. A cT4N0M0, cStage IIB (Union for International Control Cancer TNM staging) DA was diagnosed. After three courses of chemotherapy with S-1 and oxaliplatin (SOX), follow-up CT and UGE showed shrinkage of the duodenal tumor. Therefore, the patient underwent pancreaticoduodenectomy with lymph node dissection with curative intent. Histological examination showed a pathological complete response to SOX therapy. The postoperative course was uneventful, and the patient was discharged on postoperative day 29. The patient received no adjuvant chemotherapy, and there has been no evidence of recurrence 6 months after the operation.

**Conclusions:**

SOX therapy provided a remarkable response and can be an optimal chemotherapeutic regimen for advanced DA in Lynch syndrome.

## Background

Primary duodenal adenocarcinoma (DA) is a rare malignancy, representing ~ 0.5% of all gastrointestinal cancers, and is often diagnosed at an advanced stage [1–3]. Surgical resection with regional lymphadenectomy has been established as a standard treatment for DA with 5-year survival rates of 25–60% [1–3]. However, because of the relative rarity of DA, treatment guidelines or effective chemotherapeutic regimens have not been clearly defined. Furthermore, the incidence of DA is more frequent in Lynch syndrome [4], and cancers that develop in Lynch syndrome are associated with microsatellite instability (MSI). Treatment sensitivity and efficacy for tumors with MSI have not been determined [5, 6]. Here, we describe the case of an advanced DA in Lynch syndrome in which pathological complete response (pCR) was achieved with chemotherapy with S-1 and oxaliplatin (SOX).

## Case presentation

### Preoperative evaluation of the patient

A 72-year-old woman complained of right upper abdominal pain at the time of a routine check-up for colon cancer. A physical examination revealed a hard, palpable mass with pain in the middle part of the upper abdomen approximately 5 cm in diameter. Laboratory data showed an elevated leukocyte count of 10,100 cells/mm^3^ and a decreased hemoglobin level of 10.8 g/dL. Serum levels of the tumor markers carcinoembryonic antigen and carbohydrate antigen 19–9 were within normal limits. She had a history of four resections of different parts of the colon because of colon cancer associated with Lynch syndrome. At the age of 36, she was diagnosed with transverse colon cancer, and a partial resection of the transverse colon was performed. At the age of 44, she was diagnosed with cecal cancer for which ileocecal resection was performed. At the age of 45, she was diagnosed with sigmoid colon cancer, and a sigmoidectomy was performed. At the age of 72, she was diagnosed with descending colon cancer, and a partial resection of the descending colon was performed. Pathological evaluation revealed a pT2N0M0 pStage I tumor based on the seventh edition of the Union for International Cancer Control TNM staging. Her family history fulfilled the Amsterdam II and revised Bethesda criteria. Her father died of colon cancer in his 40s, one of her brothers had colon cancer at the age of 39 years, one of her cousins died of colon cancer in his 30s, and her son had ascending colon cancer at the age of 35 years; these observations suggested Lynch syndrome. After genetic counseling, a written informed consent was obtained from the patient, and we examined her for microsatellite instability (MSI). The five microsatellite markers BAT25, BAT26, NR21, NR24, and MONO27 exhibited replication errors in the descending colon cancer resected in 2017. Therefore, the patient’s colon cancer was considered to be a high-frequency MSI (MSI-high) tumor. Further genetic testing was performed using DNA from the patient’s peripheral blood. The analyses revealed one missense mutation [c.676C > T (p.Arg226)] in the MLH1 gene, thus confirming Lynch syndrome.

**Fig. 1:**
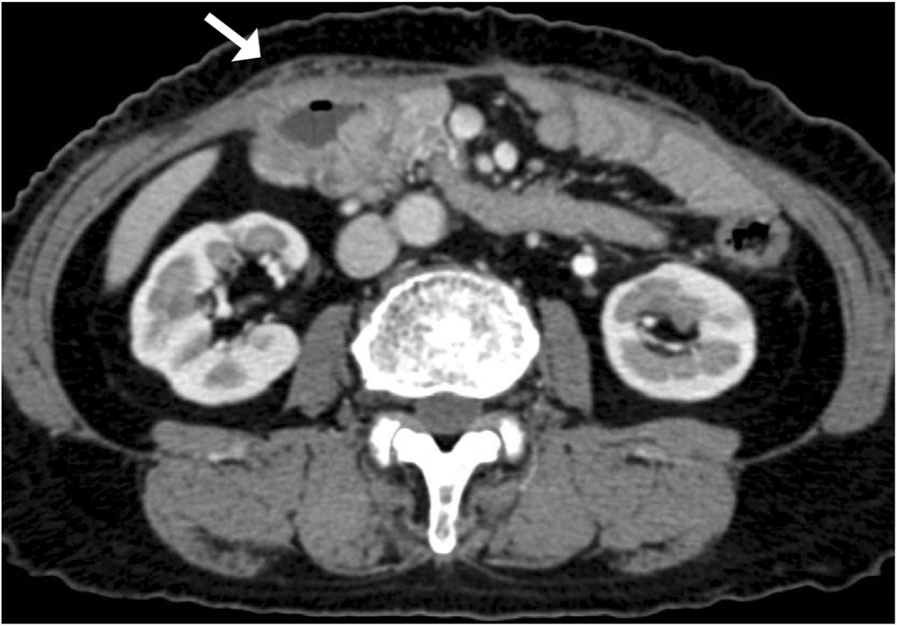
Contrast-enhanced abdominal CT scan showed wall thickness with enhancement in the second portion of the duodenum and the adjacent abdominal wall (arrow)

**Fig. 2:**
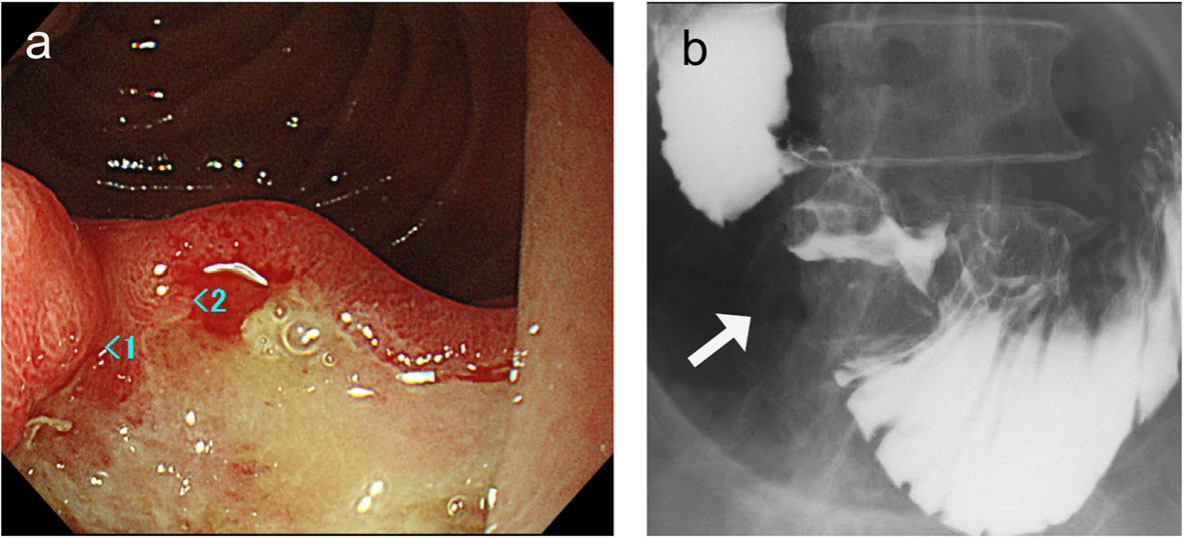
**a** Upper gastrointestinal endoscopy revealed a large, hemorrhagic, ulcerative tumor with an irregular border in the second portion of the duodenum. **b** X-ray radiography showed an ulcerative tumor 4.5 cm in diameter with an irregular border

**Fig. 3:**
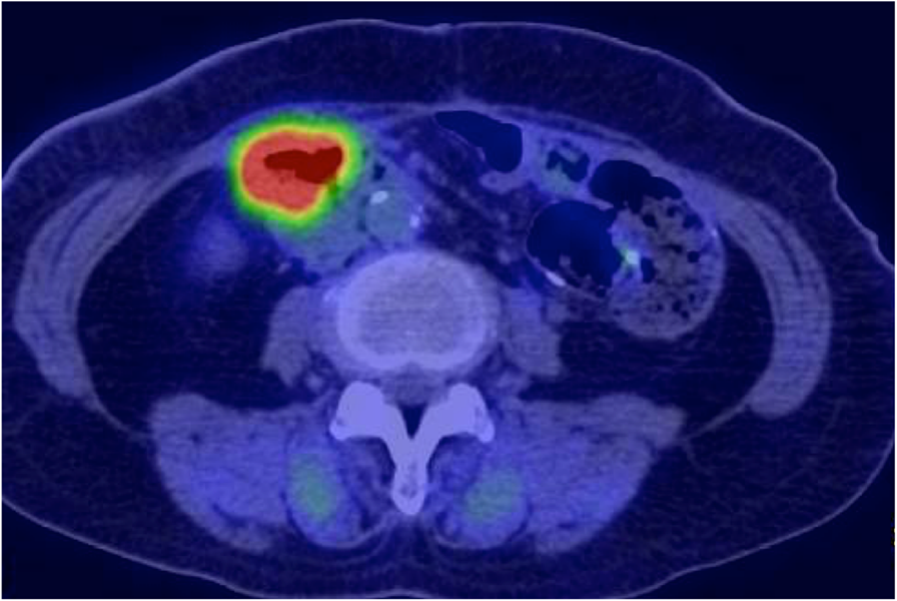
18-Fluorodeoxyglucose positron emission tomography/CT showed abnormal uptake in the tumor and widely bordering abdominal wall

Contrast-enhanced computed tomography (CT) showed wall thickness with enhancement in the second portion of the duodenum and adjacent abdominal wall, suggesting direct tumor invasion to the abdominal wall (Fig. 1). There was no regional lymph node swelling and no evidence of metastatic disease. Subsequent upper gastrointestinal endoscopy (UGE) showed a large, hemorrhagic, ulcerative tumor in the second portion of the duodenum. Histological analysis of a biopsy specimen from the tumor revealed a poorly differentiated adenocarcinoma (Fig. 2a). Upper gastrointestinal barium X-ray radiography (UGI-XR) revealed an ulcerative tumor with an irregular border measuring approximately 4.5 cm in diameter located in the second portion of the duodenum (Fig. 2b). Further imaging with 18-fluorodeoxyglucose positron emission tomography/CT demonstrated abnormal uptake in the tumor and widely bordering abdominal wall, indicating that the DA had invaded to the abdominal wall (Fig. 3). Furthermore, CT 1 month after the initial CT showed an increase in the tumor size and the abdominal wall thickness. On the basis of the above findings, the DA was clinically staged as cT4bN0M0, cStage IIB based on the seventh edition of the Union for International Cancer Control TNM staging. As the tumor had widely invaded to the abdominal wall and rapidly increased in size, the patient underwent chemotherapy to secure oncological margins.

The patient was scheduled for combination chemotherapy with SOX: 80 mg/m^2^ S-1 orally on days 1–14 and 100 mg/m^2^ oxaliplatin intravenously on day 1 of a 21-day cycle. Grade 1 adverse effects based on the National Cancer Institute Common Toxicity Criteria (version 3.0 of the toxicity scale) were neutropenia, fatigue, appetite loss, and stomatitis, all of which improved with conservative treatment.

**Fig. 4:**
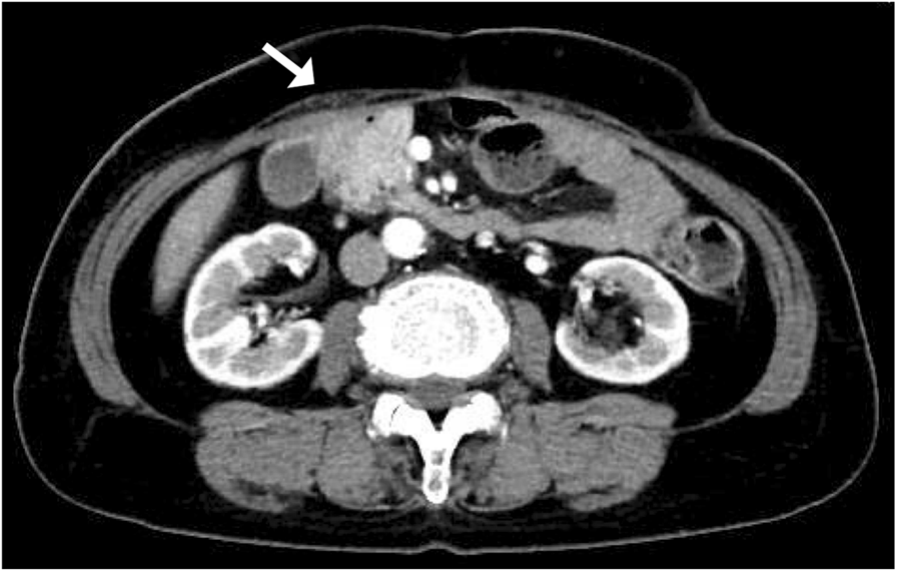
Contrast-enhanced abdominal CT after chemotherapy showed reduced duodenum tumor and abdominal wall thickness (arrow)

**Fig. 5:**
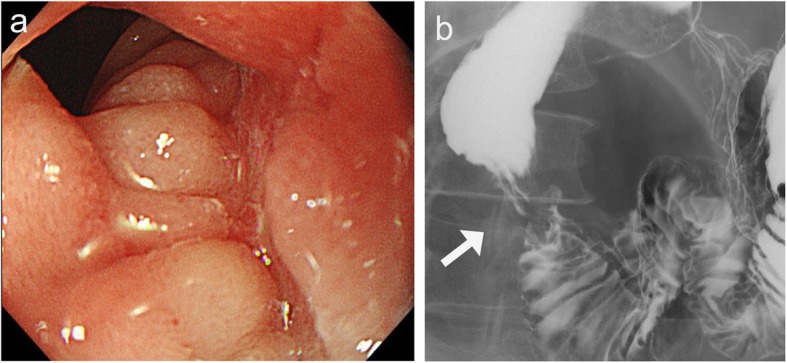
**a**, **b** Upper gastrointestinal endoscopy and X-ray radiography after chemotherapy also showed the decreased size of the ulcerative duodenal tumor (arrow)

After three courses of chemotherapy with SOX, follow-up abdominal contrast-enhanced CT revealed reduced wall thickness of the second portion of the duodenum and the adjacent abdominal wall (Fig. 4). There was no evidence of metastatic disease. UGE and UGI-XR also showed marked shrinkage of the ulcerative duodenal tumor (Fig. 5a, b).

### Operation

**Fig. 6:**
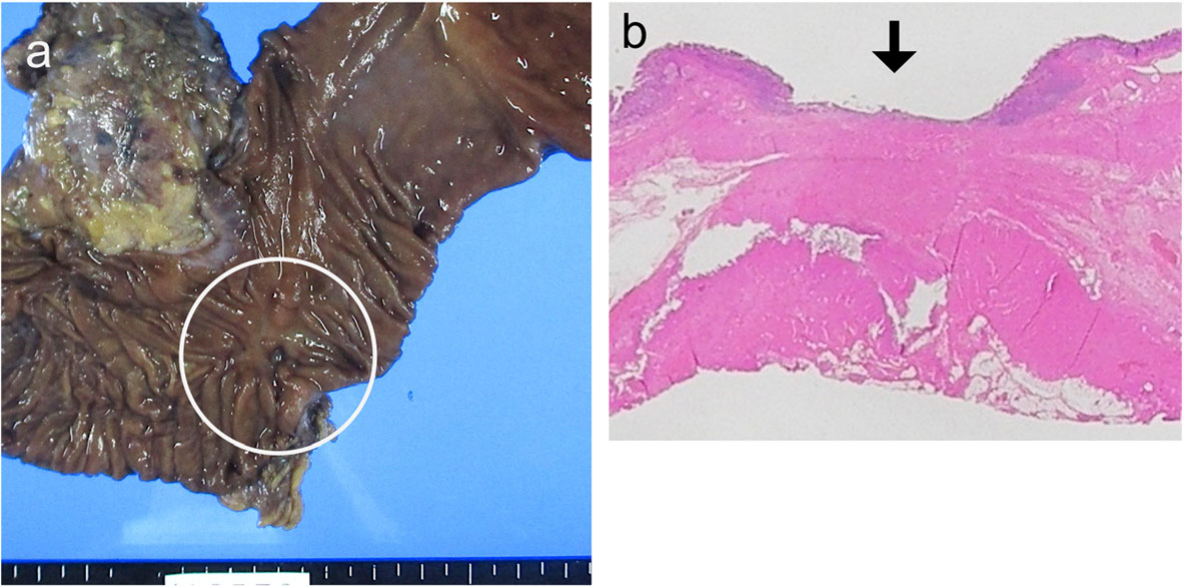
**a** Gross examination of the surgically resected specimen showed an ulcerative lesion measuring approximately 2.0 cm (circle). **b** Pathological examination of the resected specimen detected no malignant cells

The patient underwent pancreaticoduodenectomy with combined resection of the adjacent abdominal wall and regional lymph node dissection with curative intent 3 weeks after the last administration of chemotherapy. During the operation, no peritoneal dissemination or lymph node swelling was observed. Gross examination of the surgically resected specimen showed an ulcerative lesion measuring ~ 2.0 cm (Fig. 6a). Pathological examination of the resected specimen and the harvested lymph nodes detected no malignant cells. The histological effect of the chemotherapy was determined to be grade 3 according to the Japanese Classification of Gastric Carcinoma, and a pCR was diagnosed (Fig. 6b).

### Postoperative course

The postoperative course was uneventful, and the patient was discharged on postoperative day 29. The patient received no adjuvant chemotherapy, and there has been no evidence of recurrence 6 months after the operation. Postoperative surveillance is being planned according to the Japanese Society for Cancer of the Colon and Rectum Guidelines 2016 for the Clinical Practice of Hereditary Colorectal Cancer [7].

## Discussion

Primary DA is one of the rare malignancies representing ~ 0.5% of all gastrointestinal cancers, although it accounts for > 50% of small bowel adenocarcinomas (SBAs) [1, 2, 8]. Because of its non-specific symptoms and the difficulty in confirming a diagnosis, DA is often diagnosed at an advanced stage. Consequently, surgical resection was performed in 43–87% of patients [3]. Curative resection of the primary tumor has been established as a standard treatment for DA. Meijer et al. reviewed the literature and reported a 5-year overall survival (OS) rate of 46% after curative resection compared with that of 1% in palliatively treated patients [2].

DA is included in SBAs, and the outcomes for all SBAs are grouped together in many studies. Owing to the rarity of SBAs, prospective clinical trials are limited, and treatment guidelines or optimal chemotherapeutic regimens have not been clearly defined. Initially, SBAs were treated with chemotherapy based on the regimen used for gastric cancer. In 1984, Jigyasu et al. reported in a retrospective study that a 5-fluorouracil (5-FU)-based regimen for advanced SBAs achieved a response rate (RR) of 7.1% and a median OS time of 9 months [9]. Ono et al. reported that combination chemotherapy of irinotecan and cisplatin achieved an RR of 12.5% and an OS of 17.3 months [10]. In a prospective phase II study, a combination of 5-FU, mitomycin C and doxorubicin achieved an RR of 18% and a median OS of 8 months [11]. Although several studies reported improved outcomes, RR and OS remained unsatisfactory. The chemotherapeutic regimen for colorectal carcinoma could be applied to the treatment of SBA. Some studies reported that the biological characteristics or pathogenesis of SBA show higher similarity to those of colorectal cancer (CRC) than to those of gastric cancer [12–14]. Overman et al. and Xian et al. reported on CAPOX (capecitabine + oxaliplatin) therapy and FOLFOX4 (5-FU + leucovorin + oxaliplatin) therapy, and found RRs of 50% and 48.5%, median times to treatment failure of 11.3 and 7.8 months, and median OS times of 20.4 and 15.2 months, respectively [15, 16]. The addition of bevacizumab [17] or irinotecan [18] to CAPOX did not result in any significant difference in RR and progression-free survival (PFS). Thus, the combination of a fluoropyrimidine and oxaliplatin appears to be the most effective first-line regimen for unresectable small bowel cancer. The SOX regimen, the combination of the oral fluoropyrimidine derivative S-1, and oxaliplatin has been shown to be feasible and effective; therefore, it is widely used in Japan and Asia for metastatic CRC or advanced gastric cancer [19, 20].

The role of preoperative therapy for patients with locally advanced, clinically unresectable DAs has not been well documented. A retrospective study involving unresectable or recurrent DA who was treated with preoperative chemotherapy or chemoradiation found that 9 of 10 patients showed the conversion to resectable disease after the therapy, suggesting prolonged survival after conversion to resectable disease [21]. Another retrospective study demonstrated a trend toward improved 5-year survival for those patients with an R0 resection who received neoadjuvant chemoradiotherapy compared with patients who underwent surgery alone [22]. These studies have shown that preoperative therapy may be beneficial in unresectable DAs. On the other hand, several articles reported that chemotherapy for unresectable DAs achieved pCRs [23–27]. In all of the cases with pCR, the regimens used were a combination of a fluoropyrimidine and oxaliplatin, such as FOLFOX [23, 24], CapOX [26, 27], and SOX [25]. Some cases of conversion from unresectable to resectable DA by chemotherapy using a fluoropyrimidine and cisplatin have been reported. However, reported PFS and OS were poorer for a combination of 5-FU and cisplatin in comparison with those reported for a combination of 5-FU and oxaliplatin for advanced SBA [28, 29]. Accordingly, we used the combination of SOX for our patient. The latest National Comprehensive Cancer Network guidelines for small bowel adenocarcinoma recommend FOLFOX, CAPEOX, or FOLFOXIRI with/without bevacizumab for advanced or metastatic SBA including DA [30].

Lynch syndrome is a known risk factor for SBA, as are familial adenomatous polyposis, Crohn’s disease, Peutz–Jeghers syndrome, and celiac disease. In Lynch syndrome, the risk of developing SBA within a lifetime is reported to be ~ 4%, almost the same as the lifetime risk of CRC in the general population [4]. Lynch syndrome is caused by germline mutations in one of the mismatch repair genes and is associated with an increased risk of developing gastrointestinal, gynecological, and other types of cancers. The resultant deficient mismatch repair leads to MSI in cancers. Several authors reported that tumors with MSI tend to have lower sensitivity to 5-FU-based chemotherapy [5], although most studies show MSI status to be not predictive for the efficacy of chemotherapy [6]. In our case of DA, although MSI status of DA was unavailable, SOX therapy provided a remarkable response in Lynch syndrome. Furthermore, recent reports showed that a large proportion of cancers with MSI are sensitive to anti-programmed cell death protein 1 (PD-1) immune checkpoint inhibitors, regardless of cancer site or origin [31]. Since PD-1 blockade was an effective treatment for patients with SBAs [32], it can be expected to be effective for DA. The results of ongoing phase II studies with the anti-PD-1 inhibitor pembrolizumab and the anti-programmed cell death protein-1 ligand inhibitor avelumab (Clinicaltrials.gov identifier NCT02949219 and NCT03000179, respectively) for patients with refractory SBAs are also expected.

## Conclusions

We report that chemotherapy for a locally advanced DA made the surgical procedure possible and achieved pCR in Lynch syndrome. To the best of our knowledge, this is the first case of a DA patient with Lynch syndrome achieving a pCR. This case indicates that SOX therapy can be a good regimen for advanced DA even in Lynch syndrome. Since DA is a rare malignancy but occurs relatively frequently in Lynch syndrome, further clinical reports will be needed to establish the most appropriate chemotherapy regimen.

## Data Availability

None.

## Abbreviations

5-FU: 5-fluorouracil
CAPOX: Capecitabine + oxaliplatin
CRC: Colorectal cancer
CT: Computed tomography
DA: Duodenal adenocarcinoma
FOLFOX: 5-FU + leucovorin + oxaliplatin
FOLFOXIRI: 5-FU + leucovorin + oxaliplatin + irinotecan
MSI: Microsatellite instability
OS: Overall survival
pCR: Pathological complete response
PFS: Progression-free survival
RR: Response rate
SBA: Small bowel adenocarcinoma
SOX: S-1 + oxaliplatin
UGE: Upper gastrointestinal endoscopy
UGI-XR: Upper gastrointestinal barium X-ray radiography
